# Intestinal parasitic infections among children under five years of age presenting with diarrhoeal diseases to two public health facilities in Hawassa, South Ethiopia

**DOI:** 10.1186/s40249-015-0081-x

**Published:** 2015-11-04

**Authors:** Getamesay Mulatu, Ahmed Zeynudin, Endalew Zemene, Serkadis Debalke, Getenet Beyene

**Affiliations:** Department of Medical Laboratory Technology, Hawassa College of Health Sciences, Hawassa, Ethiopia; Department of Medical Laboratory Sciences and Pathology, College of Health Sciences, Jimma University, Jimma, Ethiopia

**Keywords:** Diarrhoea, Intestinal parasites, Children under 5 years, Hawassa, Ethiopia

## Abstract

**Background:**

Diarrhoea is the leading cause of morbidity and mortality in children under 5 years of age in developing countries, including Ethiopia. It is caused by a wide range of pathogens, including parasites, bacteria and viruses. The aim of this study was to determine the prevalence of infection with intestinal parasites (IPs) (and types) among children under 5 years of age with diarrhoeal diseases.

**Methods:**

A cross-sectional study was conducted at Adare Hospital and Millennium Health Centre, both located in Hawassa, South Ethiopia, from June 6 to October 28, 2011. Children under 5 years of age with diarrhoea who visited these health facilities during the study period were included in the study. Data relating to demography and risk factors associated with intestinal parasitic infections (IPIs) were gathered using a structured questionnaire. Single, fresh stool specimens were examined for IPs using the direct wet mount examination, followed by Ziehl-Neelsen staining of formol-ether concentrated samples, as per standard procedures. Data were analysed using SPSS Statistics 20 software.

**Results:**

A total of 158 children (51.3 % male and 48.7 % female) participated in the study. Overall, the prevalence of IPs was 26.6 % (42/158). Two species of IPs were detected in six children (3.8 %). *Entamoeba histolytica/dispar/moshkovskii* was the predominant parasite identified (11.4 %), followed by *Giardia duodenalis* (7.0 %). The multivariable analysis revealed that the age group ≥24 months was significantly associated (AOR = 0.221, 95 %CI: 0.085–0.576) with prevalence of IPIs.

**Conclusion:**

This study found that intestinal parasites are common among children with diarrheal diseases. The most frequently detected species was *E. histolytica/dispar*/*moshkovskii*. Health information about how to prevent diarrheal diseases in general and IPIs in particular should be provided to parents of young children.

**Electronic supplementary material:**

The online version of this article (doi:10.1186/s40249-015-0081-x) contains supplementary material, which is available to authorized users.

## Multilingual abstracts

Please see Additional file 1 for translations of the abstract into the six official working languages of the United Nations.

## Background

Globally, an estimated 1.7 billion cases of diarrhoeal disease occur each year [1]. Diarrhoea is particularly devastating for children, and remains one of the leading causes of morbidity and mortality in children under 5 years of age. Nearly one in five child deaths is due to diarrhoea [2–4] – an estimated 760,000 children die of diarrhoea annually [1].

According to the World Health Organization (WHO), diarrhoea is defined as having loose or watery stools at least three times per day, or more frequently than normal for an individual [2]. It is a common symptom of gastrointestinal infections caused by a wide range of pathogens including parasites, bacteria and viruses. In developing countries, diarrhoea is more commonly caused by intestinal parasites (IPs) and bacterial pathogens than by viruses [5].

Intestinal parasitic infections (IPIs) are the most common infections among children in developing countries. *Giardia duodenalis* (*G. duodenalis*)*, Cryptosporidium parvum* (*C. parvum*) and *Entamoeba histolytica* (*E. histolytica*) are the most common protozoan parasites that cause acute diarrhoeal illnesses in children [5]. The main clinical manifestation of infections with IPs is diarrhoea, with abdominal cramping, vomiting, flatulence and weight loss also being common symptoms. The symptoms can be severe in younger children, as well as in undernourished and immunocompromised patients. Besides protozoan parasites, intestinal helminthic infections are also a huge burden in developing countries, including Ethiopia [6–8]. Intestinal schistosomiasis and soil-transmitted helminths (STHs) are common among school children and preschool children in the country [9–11].

Ethiopia is a developing country in sub-Saharan Africa, where diarrhoea is a significant health problem in children under 5 years of age [12]. In a national survey conducted in 2011, 13 % of children under 5 years of age were reported to have had diarrhoea within two weeks of undertaking the survey, with wide variations among regions [13]. Diarrhoea is a common problem in children in the Southern Nations, Nationalities and People’s Region (SNNPR) of Ethiopia. The bacterial pathogens *Salmonella*, *Shigella* and *Campylobacter*, and the IPs *E. histolytica* and *G. duodenalis* are the common causes of diarrhoea in developing countries [14–16]. The *Cryptosporidium* species, *Isospora (Cystoisospora) belli* and *Cyclospora cayetanensis* commonly cause diarrhoea in immunocompromised patients [17]. Although bacterial etiologies of diarrhoea in children under 5 years of age in SNNPR has recently been reported [14], the burden of IPs on young children with diarrheal diseases in the region has not yet been investigated. Therefore, this study aimed to determine the prevalence of infection with IPs in children under 5 years of age with diarrhoeal diseases.

## Methods

### Study setting

This study was conducted from June 6 to October 28, 2011 in two public health facilities, namely Adare Hospital and Millennium Health Centre, in the town of Hawassa, South Ethiopia.

An estimated 250 to 300 patients visit Adare Hospital daily. The hospital provides various services, including follow-up for chronic illnesses, family planning, maternity services, emergency and inpatient services, and surgery, among others. Millennium Health Centre sees 100 to 150 patients daily. All services provided by the hospital are also available at the health centre, except for inpatient services and surgery.

**Fig. 1:**
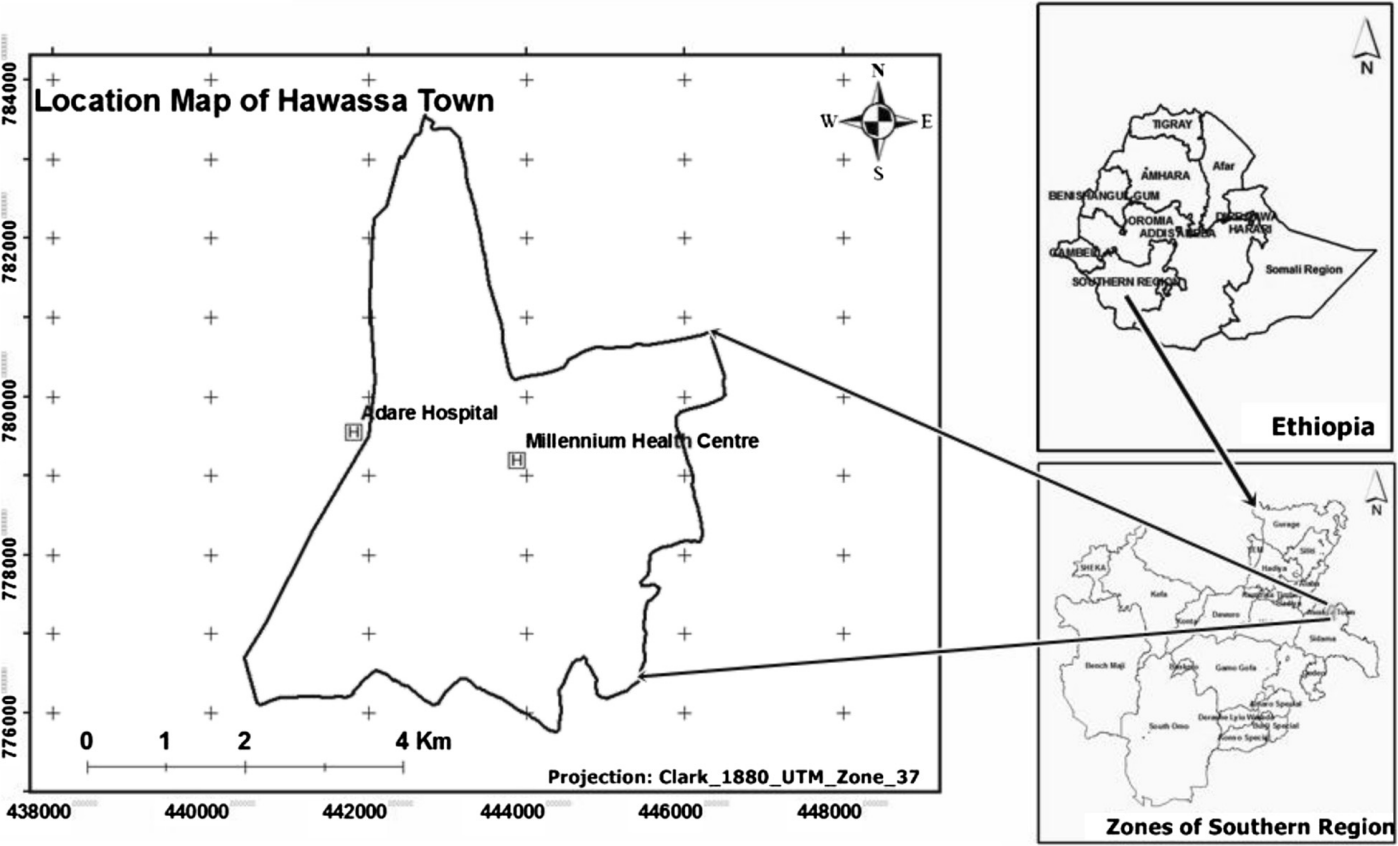
Map of the study area

Hawassa is the capital of Sidama Zone, located in the SNNPR of Ethiopia (see Fig. 1). The town is located around 270 km south of Addis Ababa, at an average altitude of 1,708 m above sea level. In 2011 (the study year), an estimated 150,000 people lived in Hawassa.

### Study participants and data collection

A total of 158 children aged three to 59 months presenting to the study health facilities with diarrhoea (at least three loose or watery bowel movements per day), whose parents/guardians gave consent to take part in the study, were included. Seventy-one children (44.9 %) were from Millennium Health Centre and 87 (55.1 %) were from Adare Hospital. The age groups of the participating children from the two health facilities were comparable.

An attending clinician collected data relating to demography and factors associated with IPIs using a pretested questionnaire. The questionnaire was first developed in English and then translated into Amharic. The children’s parents/guardians provided the socio-demographic and clinical data. In addition to the questionnaire, a stool specimen from each participating child was collected and examined for IPs.

Children who were on antiparasitic treatments and whose parents did not consent to participate in the study had to be excluded from the study. However, none of the children were on antiparasitic treatments prior to diagnosis and all parents/guardians consented to take part in the study. Hence, as all children fulfilled the inclusion criteria, no child who presented to the two health facilities with diarrhoea during the study period was excluded from the study.

### Stool specimen collection and processing

Single, fresh stool specimens were collected from study participants in clean, labelled stool cups and investigated as follows: First, direct wet mount of the fresh stool samples was microscopically examined to detect vegetative and other forms of IPs at 100x and 400x magnifications. In the direct wet mount processing, a small amount of the stool was mixed with a drop of physiological saline using an applicator stick, covered with a cover slip and examined under a microscope. Second, formol-ether concentrated specimens that were stained using the modified Ziehl-Neelsen method were examined at 1,000x magnification for detection of protozoan oocysts, namely *Cryptosporidium*, *Cyclospora* and *Isospora*. Established procedures for the direct wet mount examination, formol-ether oocyst concentration and modified Ziehl-Neelsen staining method were followed [18]. An experienced laboratory technologist processed and examined the specimens. The entire negative specimens and 10 % of the positive slides were double checked by another blinded technologist.

#### Data analysis

Data were entered into a computer, cleaned and analysed using SPSS Statistics 20 software package. Descriptive statistics were used to summarise the socio-demographic characteristics of the study participants. Bivariate and multivariable analyses were conducted to assess the risk factors associated with IPIs among the participating children. *P*-values <0.05 were considered significant.

### Ethical considerations

Ethical clearance was obtained from the Ethical Review Committee of Jimma University. Permission was obtained from Regional Health Bureau, Adare Hospital and Millennium Health Centre. Written consent was obtained from the parents/guardians of each child. Those parents/guardians who were able read and write signed the consent form themselves. Those who were unable to read and write provided their thumbprint after the information sheet and consent form was read to them. Children positive for IPs were treated at the health facilities according to national guidelines.

## Results

### Socio-demographic characteristics

**Fig. 2:**
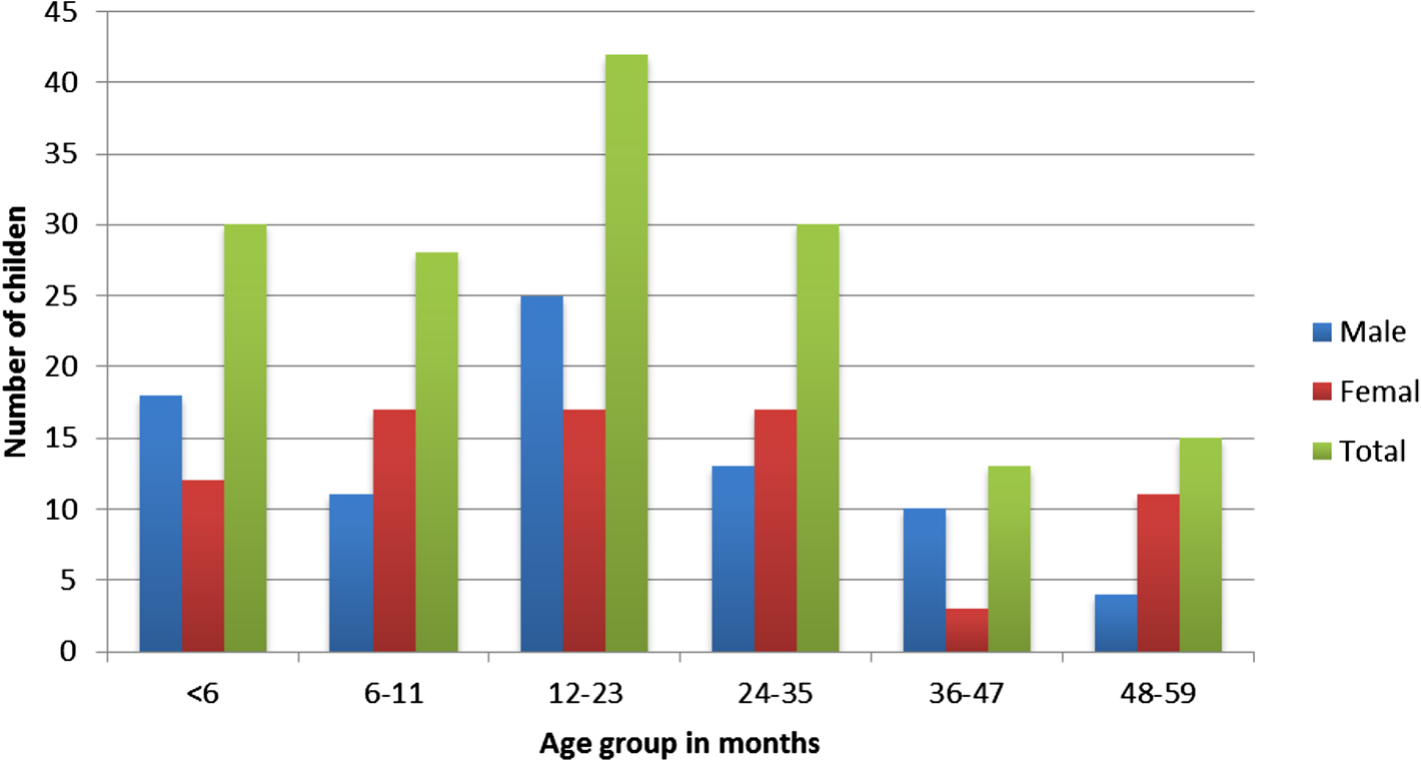
Age and sex distribution of the study participants, Hawassa, 2011

A total of 158 children aged three to 59 months presenting with diarrhoea to the two health facilities participated in the study. Eighty-seven children (55.1 %) were from Adare Hospital and 71 (44.9 %) were from Millennium Health Centre. The majority of the children (70.3 %) were aged below 24 months. Eighty-one children (51.3 %) were male. Figure 2 shows the distribution of ages and sex among the study participants.

The majority of children (91.1 %) were urban residents (residents of Hawassa) and the remaining 8.9 % were residents of the surrounding rural *kebeles* (the smallest government administrative units in Ethiopia). Most children (85.4 %) had families with five members or less.

### Prevalence of intestinal parasites

**Fig. 3:**
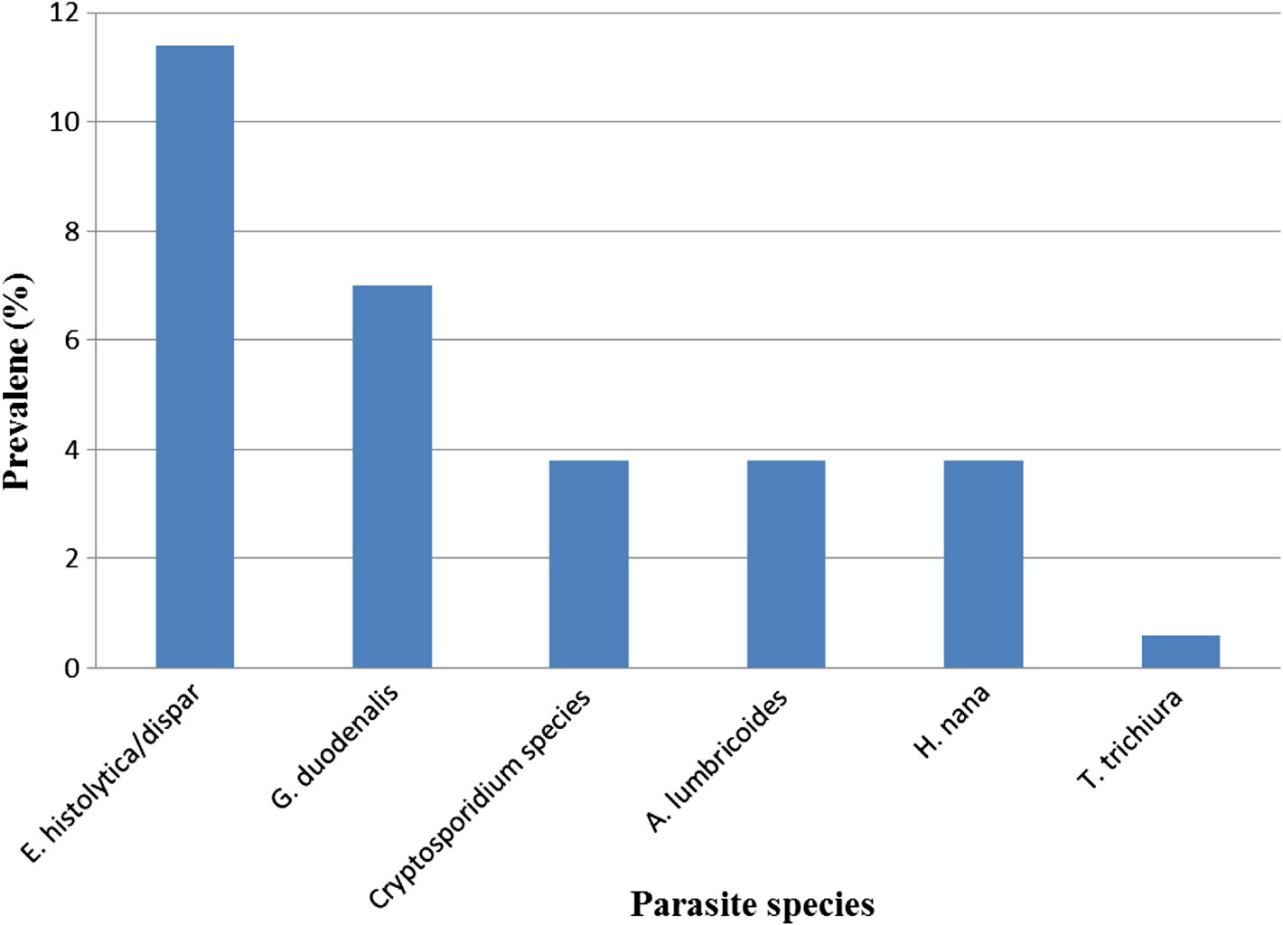
Frequency of intestinal parasites identified among children under 5 years of age with diarrhoea in Hawassa, 2011

Of the total children who participated in this study, 42 (26.6 %) were positive for at least one IP species. In six children (3.8 %), double infection was detected. *E. histolytica/dispar/moshkovskii* was the most commonly encountered parasite (11.4 %), followed by *G. duodenalis* (7.0 %). Six children (3.8 %) were also positive for the *Cryptosporidium* species. Intestinal helminths were detected in 13 children (8.2 %). *Ascaris lumbricoides* (*A. lumbricoides*) *and Hymenolepis nana* (*H. nana*) were each detected in 3.8 % of the children, while one child had *Trichuris trichiura* (*T. trichiura*). The prevalence rates of the IPs detected in this study are shown in Fig. 3.

According to the information given by the parents/guardians, more than half of the children (63.3 %) had watery diarrhoea. The duration of diarrhoea in the majority of the children (79.1 %) was 1 to 5 days. Nearly a third (32.9 %) experienced vomiting.

The prevalence of each of the IPs isolated is presented in Table 1, categorised by age group. *E. histolytica/dispar/moshkovskii* was detected in 5 (33 %) and 6 (20 %) children in the age groups 48–59 and 24–35 months, respectively*. G. duodenalis* and *Cryptosporidium* species were identified in two children and one child under 6 months of age, respectively.

**Table 1:** Distribution of intestinal parasite species by age group among children under 5 years of age with diarrhoeal diseases presenting to Adare Hospital and Millennium Health Centre, 2011, Hawassa, South Ethiopia

Age, in months	*E .histolytica/dispar/moshkovskii*		*G. duodenalis*		*Cryptosporidium* species		*A. lumbricoides/H. nana/T. trichiura*		Total
	Positive	Negative	Positive	Negative	Positive	Negative	Positive	Negative	n (%)
	n (%)	n (%)	n (%)	n (%)	n (%)	n (%)	n (%)	n (%)	
<6	0 (0)	30 (100)	2 (7)	28 (93)	1 (3)	29 (97)	0 (0)	30 (100)	30 (19)
6–11	2 (7)	26 (93)	0 (0)	28 (100)	0 (0)	28 (100)	2 (7)	26 (93)	28 (18)
12–23	3 (7)	39 (93)	1 (2)	41 (98)	0 (0)	42 (100)	2 (5)	40 (95)	42 (27)
24–35	6 (20)	24 (80)	4 (13)	26 (87)	2 (7)	28 (93)	3 (10)	27 (90)	30 (19)
36–47	2 (15)	11 (85)	2 (15)	11 (85)	0 (0)	13 (100)	4 (31)	9 (69)	13 (8)
48–59	5 (33)	10 (67)	2 (13)	13 (87)	3 (20)	12 (80)	2 (13)	13 (87)	15 (9)
Total	18 (11)	140 (89)	11 (7)	147 (93)	6 (4)	152 (96)	13 (8)	145 (92)	158 (100)

### Factors associated with intestinal parasitic infections

Factors associated with IPIs in the study participants are presented in Table 2. An almost equal proportion of male (51.3 %) and female (48.7 %) children participated in the study, with IP prevalence rates of 27.2 and 26 %, respectively. No significant difference in prevalence of IPs according to gender was observed (*p* > 0.05).

**Table 2:** Univariate and multivariable analyses of factors associated with parasitic infections among children under 5 years of age with diarrhoeal diseases presenting to Adare Hospital and Millennium Health Centre, 2011, Hawassa, South Ethiopia

Characteristics		Intestinal parasites		COR (95%CI)	AOR (95%CI)
		Positive	Negative		
		n (%)	n (%)		
Gender	Female	20 (26.0)	57 (74.0)	1	1
	Male	22 (27.2)	59 (72.8)	1.063 (0.524–2.154)	0.560 (0.233–1.344)
Age, in months	<24	18 (16.2)	93 (83.8)	1	1
	≥24	24 (51.1)	23 (48.9)	0.185 (0.087–0.398)*	0.221 (0.085–0.576)*
Residence	Urban	38 (26.4)	106 (73.6)		
	Rural	4 (28.6)	10 (71.4)	0.896 (0.265–3.028)	1.772 (0.334–9.393)
Family size	<5	34 (25.2)	101 (74.8)		
	≥5	8 (34.8)	15 (65.2)	0.631 (0.246–1.619)	0. 795 (0.235–2.693)
Breastfeeding	Yes	30 (27.7)	102 (77.3)	1	
	No	12 (46.2)	14 (53.8)	0.343 (0.124–0.821)*	0.578 (0.140–2.394)
Breastfeeding pattern	EBF	3 (6.8)	41 (93.2)	1	1
	Breastfeeding with complementary food	5 (14.3)	30 (85.7)	1.170 (0.518–2.643)	1.360 (0.55–3.366)
Complementary food initiated	After 6th month	27 (29)	66 (71)	1	1
	Before 6th month	7 (33.3)	14 (66.7)	0.801 (0.271–2.369)	0. 778 (0.234–2.589)
Contact with animals	No	33 (24.4)	101 (75.6)	1	1
	Yes	9 (37.5)	15 (62.5)	1.836 (0.735–4.585)	0.728 (0.253–2.098)

*AOR* adjusted odds ratio, *EBF* exclusive breastfeeding, *CI* confidence interval, *COR* crude odds ratio, *significant at *p* < 0.05

The IP prevalence rate among children who were exclusively breastfed (EBF) (27.8 %) and those who were breastfed but also given complementary food (22.2 %) at the point of data collection was 6.8 and 14.3 %, respectively. No significant difference was observed. The majority of the parents/guardians (83.5 %) were breastfeeding (EBF and breastfeeding with complementary food) their children at the point of data collection, and the remaining 16.5 % had ceased breastfeeding. The prevalence rates of IPs among children who were being breastfed and those who were no longer being breastfed were 30 (22.7 %) and 12 (46.2 %), respectively. A significant difference in the prevalence of IPs among the children being breastfed and those who were no longer being breastfed was observed (COR = 0.343, 95 %CI: 0.124–0.821).

The prevalence of IPs among children aged <24 months (70.3 %) and aged ≥24 months (29.7 %) was 18 (16.2 %) and 24 (51.1 %), respectively. A significant difference in the prevalence of IPs according to age group was observed (COR = 0.185, 95 %CI: 0.087–0.398). After adjusting for other variables (see Table 2), the multivariable analysis revealed that the age group ≥24 months was a predictor of IPIs (AOR = 0.221, 95 %CI: 0.085–0.576).

## Discussion

The overall prevalence of IPIs in the study participants was 26.6 %, indicating that IPs are common in children with diarrhoeal diseases in the study area. Even though the causal relationship couldn’t be established in this study, previous studies have shown that IPs are associated with diarrhoeal diseases [2, 19]. Intestinal parasitic infections may also increase susceptibility to other gastrointestinal pathogens [20].

The prevalence of IPs found in this study is lower than the prevalence reported in other studies from Gondar (52.3 %) [21] and Jimma (65.8 %) [22] in Ethiopia, and other countries such as Cameroon (59.2 %) and India (46.5 %) [23, 24]. On the other hand, the overall prevalence of IPs found in this study is slightly higher than findings from Mozambique (14.5 %), Nigeria (23.3 %) and Tanzania (15.1 %) [3, 25, 26]. These variations could be due to differences in the age, hygiene practices and parental socio-economic status of the participants involved in these studies, as well as seasonal differences. A similar prevalence of IPs was reported in a study carried out in Addis Ababa (27.5 %) [27].

The enteric protozoan pathogens *E. histolytica, G. duodenalis* and *C. parvum* are known to cause diarrhoea in children. In this study, *E. histolytica/dispar/moshkovskii* was the predominant IP isolated. Mondal et al. documented that children with *E. histolytica*-associated diarrhoeal illnesses are more likely to be malnourished and stunted [28]. Differentiations in the morphologically identical species of *Entamoeba* were not within the scope of this study, as only conventional microscopy was used to detect the amoebae. Earlier reports, however, have suggested that amebiasis was overdiagnosed in the absence of the pathogenic *E. histolytica* in adult patients in Ethiopia [29, 30].

In our study, *G. duodenalis* was the second most prevalent IP detected. Giardiasis is usually associated with poor sanitary conditions and insufficient water treatment. Nutritional deficiency is common in children symptomatically infected with *Giardia* [31]. Apart from its acute clinical symptoms, infection with *G. duodenalis* may result in cognitive deficit [32]. The *Cryptosporidium* species was detected in 3.8 % of the children in this study, which is lower than the 8.1 % prevalence reported among children with diarrhoea in Addis Ababa [27]. One *Cryptosporidium* and two *Giardia* infections were detected in infants under 6 months of age. Exposure to *Cryptosporidium* and *Giardia* in early childhood has also been reported elsewhere [33, 34].

Helminthic intestinal parasites, particularly STHs, commonly infect children in developing countries, including Ethiopia [10, 11]. In this study, helminthic infections were identified in 13 (8 %) of the children, with 7 (4.4 %) infected with the STHs *A. lumbricoides* and *T. trichiura*. The recently initiated national school-based deworming programme might have an impact on the STH infection rate among preschool children in the long run. On the other hand, as reduced efficacy of albendazole and mebendazole against *A. lumbricoides* and hookworms has been reported in Northwest Ethiopia [35], an assessment of the efficacy of anthelmintic drugs in the study area is urgently needed. Clinical symptoms signalling STH infections are related to worm burden [36]. However in this study, infection intensity was not determined. Moreover, as the single wet preparation technique was used to detect the parasites, the number of children infected with parasitic helminths could have been underestimated in this study.

In this study, 18 (16.2 %) children aged below 24 months were positive for IPs, whereas the prevalence among those aged 24 months and above was 51.1 %. This difference is significant. The higher prevalence of IPs in children aged ≥24 months as compared to children aged <24 months might be explained by the older children’s contact with faecally-contaminated soil while playing, which could predispose them to IPs.

The prevalence of IPs among EBF children and children being breastfed but also receiving complementary food was similar in this study. Exclusive breastfeeding plays a significant role in protecting against common infectious agents during infancy, as well as preventing hospitalization for diarrhoea [37]. An earlier review also indicated the role of breastfeeding in protecting against diarrhoea incidence, prevalence, hospitalizations, diarrhoea mortality and all-cause mortality [38]. The small number of children included in this study could account for the contrary results pertaining to EBF obtained – those children who were no longer being breastfed and those who were breastfed with complementary food constituted 72.2 % of the total number of study participants. Of these, 21 (18.4 %) started to receive complementary food before the age of 6 months, while 93 (81.6 %) started at the sixth month, with IP isolation rates of 33.3 and 29 %, respectively. No significant difference in prevalence rates of IPs among children who started receiving complementary food before they turned 6 months and those who received it after was observed.

In this study, the majority of the children (91.1 %) were urban residents, and living in families with less than five members (85.4 %). No significant difference in the prevalence of IPs among children from urban areas (26.4 %) and rural areas (28.6 %) was observed. Similarly, prevalence of IPs among children whose family had <5 members (25.2 %) and those whose family had ≥5 members (34.8 %) was also not significant. Other possible factors such as washing hands after defecation and whether a child wears shoes or not, and certain demographic factors of mothers, such as literacy and occupation, may affect infection of the child with IPs [39, 40]. However, these factors were not assessed in this study.

The prevalence of IPs in children with a history of contact with animals (15.2 %) and those with no history of animal contact (84.8 %) was similar. Even though intestinal protozoan parasites detected in this study are of zoonotic importance [41, 42], a limitation of this study is that data on contact with specific animals was not generated. Moreover, it has been documented that some intestinal protozoan parasites may impair the nutritional status of children [43] and diarrhoea is among the major causes of death in children with severe malnutrition [44]. However, anthropometric parameters were not measured in this study.

## Conclusion

The present study found that IPs cause diarrhoea in children under 5 years of age. Specifically, the study sheds light on the parasitic profile of children under 5 years of age with diarrhoeal diseases in Hawassa, South Ethiopia. Parasitological examinations should be considered and performed prior to empirical treatment of children under 5 years of age presenting with diarrhoea. Health information about how to prevent diarrheal diseases in general and IPIs in particular should be provided to parents of young children.

## Additional file

Additional file 1:
**Multilingual abstracts in the six official working languages of the United Nations.** (PDF 360 kb)

## Abbreviations

EBF: Exclusively breastfed
IP: Intestinal parasite
IPI: Intestinal parasitic infection
SNNPR: Southern Nations, Nationalities and People’s Region
STHs: Soil-transmitted helminths
WHO: World Health Organization
