# Supplementary material for: Intestinal parasitic infections among children under five years of age presenting with diarrhoeal diseases to two public health facilities in Hawassa, South Ethiopia
Source: Infect Dis Poverty. 2015 Nov 4;4:49. doi: 10.1186/s40249-015-0081-x (PMC4632267; doi:10.1186/s40249-015-0081-x)
Supplement: Additional file 1: — Multilingual abstracts in the six official working languages of the United Nations. (PDF 360 kb) [file 40249_2015_81_MOESM1_ESM.pdf]

Translation of the abstract into the six official working languages of the United Nations

العدوى بالطفيليات المعوية بين الأطفال الأصغر من خمس سنوات والتي تتظاهر بشكل أمراض الإسهال في اثنين من المرافق الصحية في هواسا، جنوب إثيوبيا

غيتاميساي مولاتو، أحمد زين الدين، إنداليو زيمين، سيركاديس ديبالكي، غيتينت بيين

الملخص

الخلفية: الإسهال هو السبب الرئيسي للمراضة والوفيات بين الأطفال دون سن الخامسة في البلدان النامية، ومن ضمنها إثيوبيا. وهو يحدث بسبب مجموعة واسعة من العوامل الممرضة، التي تشمل الطفيليات والبكتيريا والفيروسات. كان الهدف من هذه الدراسة تحديد مدى انتشار العدوى بالطفيليات المعوية (وأنواعها) بين الأطفال الذين تقل أعمارهم عن خمس سنوات ويعانون من أمراض الإسهال.

الطرق: أجريت دراسة عرضية (شاملة لجميع الفئات) في كل من مستشفى أدار ومركز ميلينيوم الصحي، وكلاهما يقع في هواسا، جنوب إثيوبيا، في الفترة الممتدة بين 6 يونيو - 28 أكتوبر، 2011. تم إدراج الأطفال الأصغر من خمس سنوات المصابين بالإسهال والذي زاروا هذه المرافق الصحية أثناء فترة الدراسة. تم جمع البيانات الديموغرافية وعوامل الخطر المرتبطة بالعدوى الطفيلية المعوية باستخدام استبيان مُعدّ للدراسة. تم فحص عينة براز حديثة واحدة، بحثا عن الطفيليات المعوية باستخدام الفحص الرطب المباشر، وأتبع بتلوين العينات المركزة بالفورمول-إثير بطريقة تسيل-نلسن، حسب الإجراءات القياسية. وقد تم تحليل البيانات باستخدام برنامج SPSS Statistics 20.

النتائج: شارك ما مجموعه 158 طفلا (51.3% ذكور و 48.7% إناث) في الدراسة. وبشكل عامّ، كان معدّل انتشار العدوى بالطفيليات المعوية 26.6% (158/42). تم الكشف عن نوعين من مسببات العدوى الطفيلية لدى ستة أطفال (3.8%). كان الطفيلي السائد الذي أمكن تحديده هو المتحولة الحالة للنسج /المتغيرة/موشكوفسكي *Entamoeba histolytica/dispar/moshkovskii* (11.4%)، تليه الجيارديا الإثناعشرية *Giardia duodenalis* (7.0%). وكشف التحليل متعدد المتغيرات أن الفئة العمرية ≤ 24 شهرا كان مرتبطا بشكل كبير (نسبة الأرجحية المعدلة: 0.221، 95%، مدى الثقة:0.085 – 0.576) مع مدى انتشار العدوى بالطفيليات المعوية.

الاستنتاج: وجدت هذه الدراسة أن الطفيليات المعوية شائعة بين الأطفال الذين يعانون من أمراض الإسهال. وكان أكثر الأنواع الكشف عن كثير من الأحيان هي *Entamoeba histolytica/dispar/moshkovskii*. ويجب توفير المعلومات الصحية المتعلقة بكيفية الوقاية من أمراض الإسهال بشكل عام و العدوى بالطفيليات المعوية على وجه الخصوص لوالدي الأطفال الصغار.

Translated from English version into Arabic by Lina SM, through

TRANSLATORS WITHOUT BORDERS

南埃塞俄比亚阿瓦萨两公共卫生机构中五岁以下腹泻患儿的肠道寄生虫感染情况

Getamesay Mulatu, Ahmed Zeynudin, Endalew Zemene, Serkadis Debalke, Getenet Beyene

摘要

引言：腹泻是发展中国家（包括埃塞俄比亚）五岁以下儿童发病率和死亡率的首要原因。腹泻病原体广泛，包括寄生虫、细菌和病毒。本研究旨在确定五岁以下腹泻患儿的肠道寄生虫感染（IPs）（及类型）的患病率。

方法：2011 年 6 月 6 日至 10 月 28 日，在南埃塞俄比亚阿瓦萨的阿黛尔医院和千年保健中心进行了一项横断面研究。研究对象为在此期间到这两家卫生机构就诊的五岁以下腹泻儿童。采用结构式调查问卷收集与肠道寄生虫感染（IPIs）相关的人口统计和风险因素数据。使用直接涂片法检查新鲜粪样。依据标准程序对甲醛乙醚浓缩的样品进行 Ziehl-Neelsen 染色。采用 SPSS 20 软件分析数据。

结果：共有158名儿童参加了这项研究（51.3%为男性，48.7%为女性）。IPs总患病率为26.6%（(42/158）。在6名儿童中诊断出2类IPs（3.8%）。最主要的寄生虫是溶组织内阿米巴/迪斯帕内阿米巴/莫氏内阿米巴（*Entamoeba histolytica/dispar/moshkovskii*），占11.4%，十二指肠贾第虫（*Giardia duodenalis*）占7.0%。多变量分析显示，年龄组≥24个月与IPIs的患病率显著相关（AOR=0.221，95%CI：0.085-0.576）。

结论：本研究发现，腹泻患儿往往携带肠道寄生虫。最常见的寄生虫种类为溶组织内阿米巴/迪斯帕内阿米巴/莫氏内阿米巴痢疾阿米巴/迪斯帕内阿米/莫氏内阿米巴。预防腹泻、尤其是 IPs 的相关卫生常识应提供给少儿父母。

Translated from English version into Chinese by Chen Jin, edited by Yang Pin, through

CHINESE CENTER FOR DISEASE CONTROL AND PREVENTION
NATIONAL INSTITUTE OF PARASITIC DISEASES

Infections parasitaires intestinales parmi des enfants de moins de cinq ans atteints de maladies diarrhéiques présentés auprès de deux établissements de soins de santé publics à Hawassa, Éthiopie du sud

Getamesay Mulatu, Ahmed Zeynudin, Endalew Zemene, Serkadis Debalke, Getenet Beyene

Résumé

Contexte : La diarrhée est la principale cause de morbidité et de mortalité chez les enfants de moins de cinq ans dans les pays en développement, y compris en Éthiopie. Cette affection est causée par une large variété d'agents pathogènes, dont des parasites, des bactéries et des virus. L'objectif de cette étude consiste à déterminer la prévalence de l'infection par des parasites intestinaux (PI) (ainsi que leur type) parmi des enfants de moins de cinq ans atteints de maladies diarrhéiques.

Méthodes : Une étude transversale a été menée à l'hôpital Adare Hospital et au centre de soins Millennium Health Centre tous deux situés à Hawassa, Éthiopie du sud, du 06 juin au 28 octobre 2011. Les enfants de moins de cinq ans atteints de diarrhées et ayant été présentés auprès de ces établissements de soins de santé au cours de la période de la présente étude ont été inclus à cette dernière. Les données relatives à la démographie et aux facteurs de risque associés aux infections parasitaires intestinales (IPI) ont été recueillies à l'aide d'un questionnaire structuré. La présence de PI a été examinée dans des échantillons de selles uniques et frais dans le cadre d'un examen par montage humide direct, suivi d'une coloration de Ziehl-Neelsen d'échantillons concentrés au formol-éther, conformément aux procédures standard. Les données ont été analysées à l'aide du logiciel SPSS Statistics 20.

Résultats : 158 enfants au total (51,3 % de garçons et 48,7 % de filles) ont participé à l'étude. La prévalence globale des PI s'élevait à 26,6 % (42/158). Deux espèces de PI ont été détectées chez six enfants (3,8 %). *Entamoeba histolytica/dispar/moshkovskii* a été identifié comme étant le parasite prédominant (11,4 %), suivi de *Giardia duodenalis* (7,0 %). L'analyse multivariable a révélé que le groupe d'âge > 24 mois était associé de manière significative à la prévalence d'IPI (AOR = 0,221 IC à 95 %: 0,085–0,576).

Conclusion : Cette étude a déterminé que les parasites intestinaux étaient courants parmi les enfants atteints de maladies diarrhéiques. Les espèces les plus fréquemment détectées étaient les suivantes : *E. histolytica/dispar/moshkovskii*. Des informations sur la santé relatives aux méthodes de prévention des maladies diarrhéiques de manière générale et dans le cas des IPI en particulier doivent être fournies aux parents de jeunes enfants.

Translated from English version into French by Eric Ragu, through

Интестинальные паразитарные инфекции у детей до пяти лет, представленные острыми инфекционными диарейными заболеваниями в двух государственных больницах в Аyace (Hawassa) , юг Эфиопии

Getamesay Mulatu, Ahmed Zeynudin, Endalew Zemene, Serkadis Debalke, Getenet Beyene

Фрагмент

Базовая информация. Диарея является самым распространенным и самым летальным заболеванием детей до пяти лет в развивающихся странах, включая Эфиопию. Причина диареи – широкий диапазон патогенных организмов – паразитов, бактерий и вирусов. Цель этой работы – определить распространение интестинальных паразитарных инфекций (любого типа) у детей до пяти лет (любые острые инфекционные диарейные заболевания).

Методы. Кросс-секционное, межгрупповое исследование проводилось в больнице Adare и медицинском центре Millennium, расположенных в Аyace, на юге Эфиопии, с 6 июня по 28 октября, 2011 года. Исследование охватывало детей до пяти лет, больных диареей, посетивших эти медицинские учреждения в период проведения исследования. Данные о связи желудочных паразитарных инфекций с демографическим фактором и фактором риска собирались с помощью структурированного опросника. Однократные свежие образцы стула на анализ изучались на предмет содержания интестинальных паразитарных инфекций методом анализа влажных препаратов, с последовательным окрашиванием по методу Циля-Нильсена образцов кала, прошедших *формалин-эфирную* обработку. Данные анализировались с помощью программного обеспечения SPSS Statistics 20.

Результаты. Всего в исследовании приняли участие 158 детей (51,3% мальчиков и 48,7% девочек). Присутствие интестинальных паразитарных инфекций было обнаружено у 26,6% (42/158). Два вида интестинальных паразитарных инфекций было обнаружено у шести детей (3.8%). Преобладающим патогенным организмом оказался *Entamoeba histolytica/dispar/moshkovskii* (11,4%), за ним следует *Giardia duodenalis* (7,0%). Мультивариантный анализ выявил значительную связь между возрастной группой до 24 месяцев включительно (AOR = 0.221, 95%CI: 0.085–0.576) и наличием **интестинальных паразитарных инфекций**.

Вывод. Исследование показало распространенность интестинальных паразитарных инфекций у детей, страдающих диареей. Наиболее распространенный вид организмов *E. histolytica/dispar/moshkovskii*. Родители маленьких детей должны быть снабжены детальными инструкциями по предотвращению диареи.

Translated from English version into Russian by Anna Philippova, through

Infecciones parasitarias intestinales en niños menores de cinco años que se presentan con enfermedad diarreica en dos instituciones de salud pública en Hawassa, en el sur de Etiopía.

Getamesay Mulatu, Ahmed Zeynudin, Endalew Zemene, Serkadis Debalke, Getenet Beyene

Resumen

Antecedentes: La diarrea es la principal causa de morbilidad y mortalidad en niños menores de cinco años en países en desarrollo, incluida Etiopía. Es producida por una gran variedad de agentes patógenos, que incluyen parásitos, bacterias y virus. El objetivo del presente estudio fue determinar la prevalencia de la infección por parásitos intestinales (y tipos) entre niños menores de cinco años con enfermedad diarreica.

Métodos: Se llevó a cabo un estudio cruzado en el Hospital Adare y en el Millenium Health Centre, ambos situados en Hawassa, en el sur de Etiopía, del 6 de junio al 28 de octubre del 2011. Se incluyó en el estudio a aquellos niños menores de cinco años con diarrea que visitaron estas instituciones de salud durante el período del estudio. Se utilizó un cuestionario estructurado para recolectar la información concerniente a la demografía y a los factores de riesgo asociados con infecciones parasitarias intestinales. Se examinaron muestras individuales, frescas en búsqueda de infecciones parasitarias intestinales mediante examinación directa de preparaciones en fresco, seguido de coloración Ziehl-Neelsen de las muestras concentradas en formol-éter, de acuerdo a los procedimientos estándar. La información se analizó mediante el uso del software SPSS Statistics 20.

Resultados: Un total de 158 niños (51,3% varones y 48,7% mujeres) participaron en el estudio. En general, la prevalencia de las infecciones parasitarias intestinales fue del 26,6% (42/158). Se detectaron dos especies de infecciones parasitarias intestinales en seis niños (3,8%). *Entamoeba histolytica/dispar/moshkovskii* fue el parásito predominantemente identificado (11,4%), seguido de *Giardia duodenalis* (7,0%). El análisis multivariable reveló que el grupo de edad $\geq$24 meses estaba significativamente relacionado (AOR=0,221, 95% IC: 0,085–0,576) con prevalencia de las infecciones parasitarias intestinales.

Conclusión: Este estudio encontró que los parásitos intestinales son comunes en los niños con enfermedades diarreicas. Las especies más frecuentemente detectadas fueron *E. histolytica/dispar/moshkovskii*. Se debería proporcionar a los padres de niños pequeños información médica sobre cómo prevenir las enfermedades diarreicas en general y las infecciones parasitarias intestinales en particular.

Translated from English version into Spanish by Maria Alejandra Aguada, through

TRANSLATORS WITHOUT BORDERS
